# Selected Cytokines in Patients with Pancreatic Cancer: A Preliminary Report

**DOI:** 10.1371/journal.pone.0097613

**Published:** 2014-05-21

**Authors:** Wojciech Błogowski, Anna Deskur, Marta Budkowska, Daria Sałata, Anna Madej-Michniewicz, Krzysztof Dąbkowski, Barbara Dołęgowska, Teresa Starzyńska

**Affiliations:** 1 Department of Public Health, University of Zielona Góra, Zielona Góra, Poland; 2 Department of Gastroenterology, Pomeranian Medical University in Szczecin, Szczecin, Poland; 3 Department of Medical Analytics, Pomeranian Medical University in Szczecin, Szczecin, Poland; University of North Carolina School of Medicine, United States of America

## Abstract

**Background/Aims:**

Recent experimental studies have suggested that various cytokines may be important players in the development and progression of pancreatic cancer. However, these findings have not yet been verified in a clinical setting.

**Methods:**

In this study, we examined the levels of a broad panel of cytokines, including interleukin (IL)-1, IL-6, IL-8, IL-10, IL-12, IL-17, and IL-23, as well as tumor necrosis factor alpha (TNFα) and granulocyte-colony stimulating factor (G-CSF) in patients with pancreatic adenocarcinoma (n = 43), other pancreatic malignancies (neuroendocrine [n = 10] and solid pseudopapillary tumors [n = 3]), and healthy individuals (n = 41).

**Results:**

We found that there were higher levels of IL-6, IL-8, IL-10 and TNFα in patients with pancreatic cancer compared to healthy controls (for all, at least p<0.03). Cancer patients had lower IL-23 concentrations than healthy individuals and patients diagnosed with other types of malignancies (for both, p = 0.002). Levels of IL-6, IL-8, IL-10, and IL-23 were significantly associated with the direct number of circulating bone marrow (BM)-derived mesenchymal or very small embryonic/epiblast-like stem cells (SCs) in patients with pancreatic cancer. Moreover, our study identified a potential ability of IL-6, IL-8, IL-10, IL-23, and TNFα levels to enable discrimination of pancreatic cancer from other pancreatic tumors and diseases, including acute and chronic pancreatitis and post-pancreatitis cysts (with sensitivity and specificity ranging between 70%–82%).

**Conclusions:**

Our study i) supports the significance of selected cytokines in the clinical presentation of pancreatic cancer, ii) highlights numerous associations between selected interleukins and intensified BMSCs trafficking in patients with pancreatic cancer, and iii) preliminarily characterizes the diagnostic potential of several cytokines as potential novel clinical markers of pancreatic cancer in humans.

## Introduction

Pancreatic cancer is one of the most fatal gastrointestinal malignancies. It occurs in Western societies worldwide, with an incidence that is increasing at an alarming rate [1], [2]. Several risk factors for this malignancy have been identified, including male gender, advanced age, obesity, chronic pancreatitis, a family history of pancreatic cancer, and genetic predisposition [1], [3]. However, despite extensive scientific and clinical efforts to elucidate the pathogenesis of this disease, the exact molecular mechanisms responsible for the initiation, development, and progression of pancreatic cancer in humans are still poorly understood.

Recently, there has been increased interest in the potential involvement of stem cells (SCs) in pancreatic cancer. Several research teams have already used experimental animal models to demonstrate the existence of a population of SCs, termed pancreatic cancer stem cells (PCSCs), that seem to be responsible for the initial development of pancreatic cancer as well as the promotion of its systemic spread [4]–[9]. Our team recently expanded these observations by reporting that patients with pancreatic cancer exhibit intensified systemic trafficking of various populations of bone marrow (BM)-derived SCs (BMSCs). This trafficking is mainly of Lin^-^CD45^-^CD133^+^CXCR4^+^ very small embryonic/epiblast-like SCs (VSELs) and CD45^-^CD90^+^CD105^+^ mesenchymal SCs (MSCs) [10]. However, little is known about the pathological mechanisms that are responsible for this phenomenon. Surprisingly, various studies in patients with pancreatic cancer showed that systemic circulation of SCs is not associated with increased levels of SC chemoattractants, such as stromal-derived factor 1α. Instead, this systemic circulation of SCs is linked to the action of the innate immune system, mainly the complement cascade [10], [11]. Nevertheless, other immunological factors could very likely be involved in the regulation of this process, and this should be verified in clinical studies.

Cytokines are excellent candidate factors for the orchestration of both BMSC mobilization and the promotion of pancreatic cancer development in humans. For example, studies have shown that various cytokines may promote formation of blood vessels within the pancreatic tumor microenvironment, can independently and/or synergistically influence the activity of immune cells, the intensity of inflammatory processes, and the invasiveness of pancreatic cells, therefore contributing to metastatic spread [12]–[19].

Taking all these facts into consideration, in this study, we decided to comprehensively analyze systemic levels of the previously noted cytokines in patients with pancreatic cancer and compare cytokine levels among pancreatic cancer patients, healthy individuals, and patients with other pancreatic malignancies. We also verified our previous observations concerning associations between systemic levels of cytokines and absolute numbers of circulating BMSCs in patients with pancreatic cancer (previously reported by us [10]). In this study, we also determined whether systemic levels of cytokines have clinical value for the diagnosis and discrimination of pancreatic cancer from other pancreatic tumors and diseases in humans. We hypothesized that patients suffering from pancreatic cancer would have higher levels of certain cytokines, and that these elevated levels would be at least partially associated with intensified systemic trafficking of BMSCs. We also posited that cytokine levels could potentially serve as novel markers for distinguishing pancreatic cancer from other pancreatic tumors and diseases in humans.

## Materials and Methods

### Ethics statement

The Bioethical Committee of the Pomeranian Medical University in Szczecin approved the study protocol, and all patients provided written informed consent prior to participation.

### Patients and blood samples

A total of 142 individuals with generally good and stable health were included in the study. These patients were divided into groups: a “cancer” group (newly diagnosed pancreatic adenocarcinoma, n = 43), an “other malignancies” group (pancreatic neuroendocrine tumors [NETs], n = 10; solid pseudopapillary tumors [SPTs], n = 3), an “other pancreatic diseases” group (acute/chronic pancreatitis, n = 31; pancreatic cysts, n = 14), and a “control” group (healthy volunteers, n = 41).

The final diagnosis of pancreatic adenocarcinoma and other malignancies was based on biopsy specimen analysis. In order to establish disease staging, all patients underwent ultrasonography, computed tomography and/or endoscopic ultrasonography, as well as chest x-ray examinations. In the “cancer” group, 6 patients qualified for surgical removal of the pancreatic tumor (Stage I or II according to the Tumor-Node-Metastasis – TNM classification), 10 patients presented with inoperable, locally advanced disease (Stage III), and 27 patients had distal metastases to the liver or lungs (Stage IV). At the time of their inclusion in the study, none of the patients was on chemotherapy treatment, had received any cytotoxic agents or drugs within the last 12 months before the study, or had presented any clinical signs of an active infectious disease. All patients were recruited from individuals hospitalized in the Department of Gastroenterology of the Pomeranian Medical University in Szczecin. The general characteristics of the individuals enrolled in the study, together with a statistical comparison of these features between the examined groups, are presented in Table 1 and Table S1.

**Table 1:** General characteristics of analyzed patients and healthy individuals enrolled in the study (data presented as means ± SD or median [interquartile range]).

Parameters	control group	Cancer	other malignancies
Age (years)	60±7	61±9	48±18
Gender (M-men/W-women)	15-M/26-W	17-M/26-W	6-M/7-F
BMI (kg/m^2^)	25.69±3.57	22.79±7.24	23.99±3.47
RBC (x10^12^ cells/L)	4.77±0.58	4.17±1.03	4.74±0.38
Hb (g/dL)	14.08±1.74	12.71±1.60*	13.78±0.79
Platelets count (x10^9^ cells/L)	224±62	264±109	231±48
WBC count (x10^9^ cells/L)	6.08±1.75	8.82±3.50	6.84±2.12
CRP (mg/L)	2.91±1.84	13.40 [4.80; 70.20]**#	3.00 [1.85; 7.00]
CA19.9 (U/mL)	12.41±5.27	494.12 [112.35; 1675.75]**	9.48 [3.73; 15.14]**

BMI – body mass index RBC – red blood cells Hb – hemoglobin.

WBC – white blood cells CRP – C-reactive protein.

*P<0.05 and **P<0.01 (vs control group).

# P<0.05 (vs other pancreatic malignancies group).

Peripheral blood samples (8–10 mL) were collected from all included individuals. Samples were processed immediately according to standard laboratory protocols, and plasma was separated, frozen, and stored at −80°C until further assessment.

### Analysis of systemic levels of cytokines

The systemic concentrations of interleukins (IL-1, IL-6, IL-8, IL-10, IL-12, IL-17, and IL-23), G-CSF, and TNFα were measured using commercially available, high-sensitivity ELISA kits (*R&D Systems, Minneapolis, MN, USA* or *BD Bioscience OptEIA ELISA Kits, MD, USA*) according to the manufacturer instructions.

### Statistical Methods

Analogically as in our previous studies [20]–[24] the Shapiro–Wilk test was used to determine the distribution of the continuous variables analyzed. The Student's t-test was used to compare mean parameter values between the examined groups (for normally distributed variables). For variables that were not normally distributed, the values were log transformed. If a normal distribution was then achieved, these transformed variables were also compared using the Student's t-test. However, if the transformation did not result in a normal distribution, a Mann-Whitney U-test was performed. Correlations between various analyzed parameters were calculated using the Pearson test or Spearman rank test, according to the normality of the distribution. To evaluate the effects of continuous variables on pancreatic cancer staging and levels of cytokines, multivariate regression analyses were performed using a stepwise selection method. Variables excluded from the initial model were re-entered individually to exclude residual confounding. During development of multivariate regression models, the number of inserted independent variables did not exceed 10% of the total number of analyzed patients. Constructed models were verified using the Akaike information criterion (AIC), and wrongly constructed matrices resulted in rejection of the model. Receiver operating characteristic (ROC) curves were constructed for all parameters analyzed as diagnostic for pancreatic cancer, and the area under each ROC curve (AUC) was calculated. The current study has been performed in accordance to the REMARK guidelines to improve the quality and general validity of its results. Statistical analysis was performed using SPSS statistical analysis software. P-values less than 0.05 were considered significant.

## Results

### Analysis of included individuals

Initial comparison of the analyzed groups of recruited individuals revealed significantly higher CA19-9 levels in patients with pancreatic adenocarcinoma compared to all other patient groups (Table 1 and Table S1). Moreover, cancer patients had significantly higher C-reactive protein levels than control individuals and subjects diagnosed with other types of pancreatic malignancies, but these values were lower than those detected in patients with other pancreatic diseases, and did not significantly differ between patients diagnosed with early and more advanced disease (Table S2).

### Comparison of systemic levels of cytokines in patients with pancreatic cancer, other pancreatic malignancies, and healthy individuals

**Figure 1:**
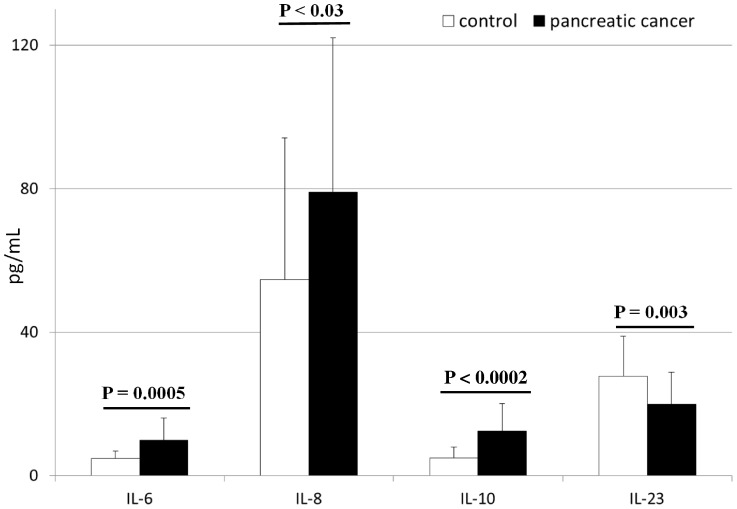
Levels of selected interleukins in patients with pancreatic cancer and healthy individuals together with their statistical comparison (means ± standard deviation). IL – interleukin p – level of significance.

**Figure 2:**
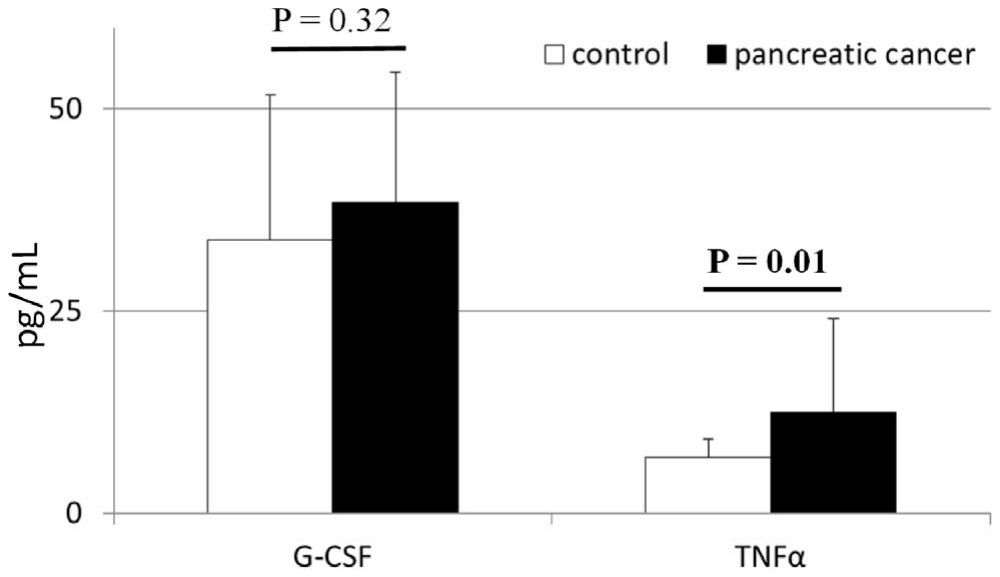
Levels of selected cytokines in patients with pancreatic cancer and healthy individuals together with their statistical comparison (means ± standard deviation). G-CSF – granulocyte-colony stimulating factor TNFα – tumor necrosis factor alpha p – level of significance.

The mean systemic concentrations of interleukins in patients with pancreatic adenocarcinoma and healthy individuals are depicted in Figure 1 and Figure S1. We found that levels of IL-6, IL-8, and IL-10 were significantly higher (up to 3 times) in cancer patients than in healthy individuals. In contrast, IL-23 concentrations were significantly lower in cancer patients than in healthy controls (Figure 1). We observed no significant differences in IL-1β, IL-12, IL-23, or G-CSF levels between healthy individuals and cancer patients (Figure 2 and Figure S1). In addition, patients with pancreatic adenocarcinoma had G-CSF levels that were similar to healthy individuals, and significantly higher TNFα concentrations (Figure 2). Interestingly, when we compared these cytokines levels between patients with early diagnosed, locally advanced and metastatic disease, we found statistically significant differences only in terms of IL-8 concentrations (Table S2).

Interestingly, when we compared levels of cytokines between healthy individuals and patients with other pancreatic malignancies, the only difference was in the concentration of IL-23 (Table S3). In addition, systemic levels of IL-23 in patients with NETs or SPTs were significantly higher than levels in pancreatic cancer patients (p = 0.002). Importantly, patients with pancreatic adenocarcinoma had significantly higher systemic IL-8 and TNFα levels than did individuals diagnosed with other pancreatic malignancies (Table S3).

### Cytokines and circulating bone marrow-derived stem cells

After we identified the cytokines that differ between cancer patients and healthy individuals, we were interested in whether levels of these cytokines are associated with the recently reported phenomenon of intensified systemic circulation of various populations of BMSCs in patients with pancreatic adenocarcinoma [10]. We found that levels of IL-6, IL-8, IL-10, and IL-23 significantly correlated with the absolute number of circulating BM-derived MSCs, and IL-23 levels were also negatively associated with the number of circulating VSELs (Table S4). There were no other significant associations between systemic levels of cytokines and the absolute number of systemically circulating cells from other populations of BMSCs.

### Clinical associations between cytokines and pancreatic cancer

Finally, we verified potential associations between systemic levels of cytokines and the clinical presentation of pancreatic malignancy in our patients. Using multivariate regression analyses we found that, among all examined cytokines, only IL-8 levels in patients with pancreatic cancer were strongly associated with disease advancement. The IL-8 concentration also seemed to be associated with or influenced by IL-6 and TNFα levels in patients with pancreatic adenocarcinoma (Table 2).

**Table 2:** Analysis of associations between levels of examined cytokines and clinical presentation of pancreatic cancer in patients (modelling using multivariate regression analysis).

Dependent variable	Independent variable(s)	β [95% CI]	P of the variable	R^2^	P of the model
*Disease progression* *	IL-8	0.71 [0.61; 0.80]	0.0008	0.49	0.0008
*IL-8*	IL-6	0.50 [0.42; 0.57]	0.0006	0.27	0.0006
	TNFα	0.09 [0.05; 0.13]	0.04	0.07	0.04

β – standardized coefficient in the regression equation p – level of significance.

IL – interleukin TNFα – tumor necrosis factor alpha.

*early resectable disease, locally invasive disease and metastatic spread were assigned 0, 1 and 2 value (respectively).

**Figure 3:**
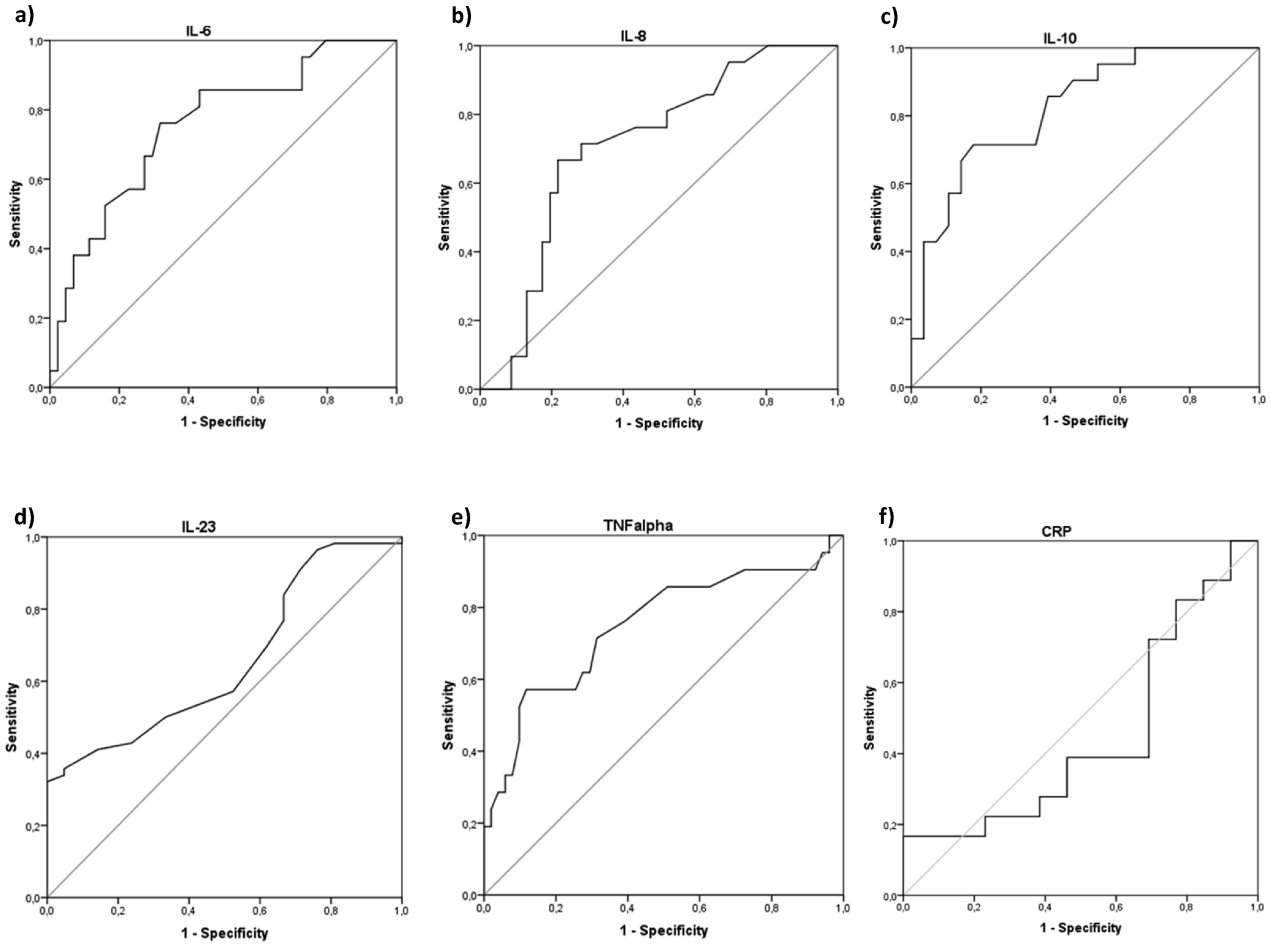
Receiver operating characteristics (ROC) curves of interleukins/cytokines and C-reactive protein as indicators of pancreatic cancer or non-cancerous lesions. Calculated sensitivity (y-axis) is plotted against 1-specificity formula (x-axis) for examined interleukins/cytokines, that is IL-6 (a), IL-8 (b), IL-10 (c), TNFα (e), and C-reactive protein (f) as indicators of pancreatic cancer, and IL-23 (d) as a marker of non-cancerous lesions. Precise description of these parameters (a-e) is presented in Table 3. IL - interleukin TNFα – tumor necrosis factor alpha p – level of significance CRP – C-reactive protein.

After noting such evident differences in the levels of selected cytokines between the examined groups of patients and healthy individuals, we decided to preliminarily examine the potential diagnostic value of these selected cytokines for the detection of pancreatic cancer in humans. To determine whether systemic levels of cytokines could serve as novel makers of pancreatic adenocarcinoma, we added a group of patients who were hospitalized or diagnosed with other pancreatic diseases (acute/chronic pancreatitis or pancreatic cysts) to our analyses. After measuring cytokine levels (IL-6, IL-8, IL-10, IL-23, TNFα) in this group, we added these patients to our analyses, constructed ROC curves, and determined the approximate AUC values to assess the suitability of these cytokines as diagnostic markers for pancreatic cancer. Among all examined parameters, only those with a 95% confidence interval (CI) lower bound value that exceeded 0.50 are presented and precisely described (Figure 3). Our analysis demonstrated that IL-6, IL-8, IL-10, and TNFα are promising novel candidate markers for the detection of pancreatic adenocarcinoma, whereas IL-23 might be valuable for its ability to exclude a diagnosis of cancer. Based on our results, we decided to determine suggested diagnostic cut-off values for these cytokines and preliminarily characterize their estimated sensitivity, specificity, and positive and negative predictive values (Table 3). Interestingly, in our analysis C-reactive protein levels seemed to be of no diagnostic value for indication of pancreatic cancer (AUC = 0.45; 95% CI 0.24–0.66; p = 0.63) (Figure 3f), and currently used CA19.9 marker proved to be of 74.4% sensitivity, 80.8% specificity, while its positive and negative predictive values were 62.7% and 87.9%, respectively.

**Table 3:** Diagnostic value of examined cytokines to discriminate pancreatic cancer from non-cancerous states in humans.

Parameter	IL-6	IL-8	IL-10	IL-23	TNFα
**Indication of**	Cancer	cancer	cancer	other malignancies	cancer
**Area under ROC curve**	0.82; p<0.0002	0.71; p<0.01	0.82; p<0.0001	0.65; p<0.04	0.74; p<0.005
**Suggested cut-off value**	≥4,92 pg/mL	≥51,15 pg/mL	≥7,35 pg/mL	≥32,5 pg/mL	≥6,75 pg/mL
**Sensitivity** [%]	86.0	72.1	72.1	34.9	76.7
**Specificity** [%]	56.6	71.7	81.8	94.9	60.6
**Positive predictive value** [%]	46.3	52.5	63.3	75.0	45.8
**Negative predictive value** [%]	90.3	85.5	87.1	77.0	85.7

IL – interleukin TNFα – tumor necrosis factor alpha.

ROC – receiver operating characteristics p – level of significance.

## Discussion

For many years, researchers have been highlighting the fact that biochemical and molecular crosstalk between immune and cancer cells is crucial for systemic progression of malignancies. So far, several experimental studies have demonstrated a significant role of various cytokines, together with growth factors, in pancreatic cancer development (reviewed in detail in [25], [26]). However, very few of these observations have been confirmed in an actual clinical setting. Therefore, we decided to comprehensively analyze a wide panel of cytokines in patients with pancreatic cancer. We used these data to verify potential associations of cytokines with systemic BMSC circulation and to evaluate the potential clinical diagnostic value of cytokine levels.

We found that, among all analyzed cytokines, only IL-6, IL-8, IL-10, and IL-23 levels significantly differed between patients with pancreatic cancer and other individuals. From a molecular standpoint, elevations in the levels of these ILs may strongly promote cancer progression in patients with pancreatic malignancies, as these cytokines are involved in the activation of multiple upstream signaling pathways that influence the activity of numerous transcription factors, modulate the cellular proteome at both the genetic and translational level, and promote the development of an “immunosuppressive” Th2 immune phenotype profile [12]–[14], [27]–[29]. Surprisingly, we did not see significant differences in the levels of other ILs that we examined, which are known to stimulate immune response [17], [19], [30] and could beneficially promote immune aggression against cancer cells leading to their successful elimination. All these results confirm and translate previous observations, which showed that not only a local, but also a systemic endogenous “immunosuppressive” state seems to occur during the development and progression of pancreatic adenocarcinoma in humans [31], [32]. It seems also very intriguing to define the exact (patho)mechanisms that lead to such specific constellation of cytokines in patients with pancreatic cancer. It is suggested that such phenomenon may be related to multiple factors, including strong desmoplastic reaction from the cancer environment that seems to be a hallmark in pancreatic cancer. Several types of tumor-stroma interactions have been implicated as having the potential to promote pancreatic cancer cell invasion and metastasis. Researchers have demonstrated that the environment of developing pancreatic cancer contains multiple cells, such as fibroblasts, pancreatic stellate cells, aberrant endothelial cells, pericytes, foci of inflammatory cells and macrophages that may strongly contribute to production of chemokines and cytokines (reviewed in detail in [33]). Moreover, on the systemic level various types of cells may also contribute to generation of cytokines including bone marrow-derived stem cells, as well as, immune cells, such as CD4+ T lymphocytes or dendritic cells [34], [35]. All these mechanisms may simultaneously lead to an altered cytokine profile in patients with pancreatic cancer, and thereby promote its development and systemic spread. Interestingly, suppression of various cytokines (for example IL-17) leads to significant inhibition of (pancreatic) tumor growth in experimental animals [36]. This effect is believed to be mainly related to inhibited metalloproteinases' and VEGF action, as well as, promotion of a “Th1-dominant pro-inflammatory” environment. Nevertheless, further studies are undoubtedly needed to fully examine and understand this process in humans.

We also demonstrated that elevated levels of certain ILs in patients with pancreatic cancer are associated with the absolute number of circulating BMSCs, mainly MSCs. Recently, we reported that selective mobilization of BMSCs occurs in patients with pancreatic adenocarcinoma, and various populations of BMSCs such as hematopoietic stem and progenitor cells (HSPCs) or endothelial progenitor cells (EPCs) seem to be repressed within the BM environment, while VSELs and MSCs egress from the BM [10]. In this study, we surprisingly found that G-CSF was not significantly associated with BMSC trafficking in patients with pancreatic cancer, as its levels were comparable between examined groups. For many years, G-CSF was expected to be associated with BMSC trafficking in pancreatic cancer patients, although our report that it is not significantly associated confirms more recent reports by others [37], [38]. Additionally, our results demonstrated that selected ILs might indeed be potentially involved in the regulation of this process. Particularly, IL-6, IL-8, IL-10, and IL-23 concentrations in pancreatic cancer patients seem to be associated with the number of circulating MSCs, whereas lower IL-23 levels negatively correlated with increased numbers of VSELs detected in the peripheral blood of patients diagnosed with pancreatic adenocarcinoma. Unfortunately, at this stage of our research, it is impossible to define the exact role of these ILs in the regulation of BMSC homeostasis or trafficking in pancreatic cancer patients. This aspect should be fully examined in future experimental and translational studies.

Finally, we decided to conduct a preliminary analysis of the potential diagnostic value of specific cytokines for the detection of pancreatic cancer, and for the discrimination of pancreatic cancer from other pancreatic malignancies and diseases. Several researchers are constantly trying to discover novel substances that could be routinely used in clinical practice as markers of pancreatic cancer [37]–[39]. Thus far, these attempts have met with variable success. In our study, we found that systemic levels of IL-6, IL-8, IL-10, and TNFα show potential as diagnostic markers for the detection of pancreatic adenocarcinoma (with sensitivity and specificity of approximately 70–80%), whereas IL-23 concentrations could potentially be used to exclude a diagnosis of pancreatic cancer in humans. Unfortunately, even though our preliminary results are very promising, at this stage, these markers do not seem suitable for independent decision making because of their relatively low specificity and because their diagnostic value was determined on the basis of a relatively small sample size. In addition, the suggested cut-off and predictive values of these markers should be further verified in cohort studies.

In summary, our clinical study i) supported the significance of selected cytokines in the clinical presentation of pancreatic cancer in humans, ii) highlighted several associations between selected ILs and intensified BMSC trafficking in patients with pancreatic adenocarcinoma, and iii) preliminarily characterized the diagnostic potential of several cytokines as potential novel clinical markers for pancreatic cancer in humans.

## Supporting Information

Figure S1 **Levels of selected interleukins in patients with pancreatic cancer and healthy individuals together with their statistical comparison (means ± standard deviation).**
(PDF)

Table S1 **General characteristics of patients diagnosed with acute/chronic pancreatitis or pancreatic cysts (data presented as means ± SD or median [interquartile range]).**
(PDF)

Table S2 **Cytokine and C-reactive protein levels in pancreatic cancer patients, subdivided into groups according to the Tumor-Node-Metastasis (TNM) staging of malignancy (presented as means ± SD or medians [interquartile range]).**
(PDF)

Table S3 **Levels of examined cytokines in patients with other malignancies (NETs and SPTs) together with their statistical comparison (medians [interquartile range]).**
(PDF)

Table S4 **Coefficients of correlations between absolute numbers of circulating bone marrow-derived stem cells' populations and systemic levels of examined cytokines in patients with pancreatic** ***adenocarcinoma*** **(n = 8).**
(PDF)
