# Supplementary material for: Selected Cytokines in Patients with Pancreatic Cancer: A Preliminary Report
Source: PLoS One. 2014 May 21;9(5):e97613. doi: 10.1371/journal.pone.0097613 (PMC4029741; doi:10.1371/journal.pone.0097613)
Supplement: Figure S1 — Levels of selected interleukins in patients with pancreatic cancer and healthy individuals together with their statistical comparison (means ± standard deviation). (PDF) [file pone.0097613.s001.pdf]

Figure S1. Levels of selected interleukins in patients with pancreatic cancer and healthy individuals together with their statistical comparison (means ± standard deviation).

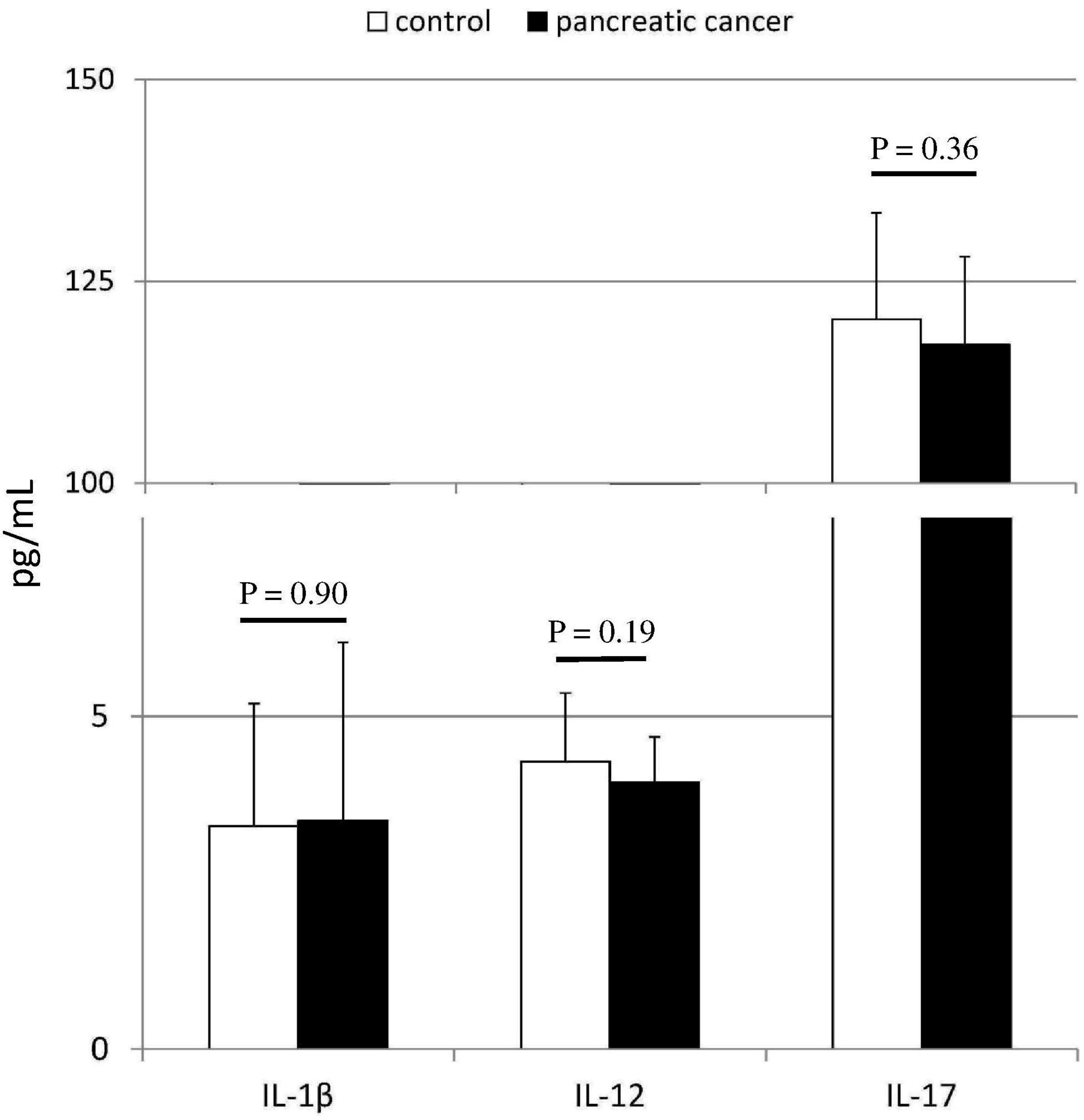

IL – interleukin

p – level of significance
