# Supplementary material for: Selected Cytokines in Patients with Pancreatic Cancer: A Preliminary Report
Source: PLoS One. 2014 May 21;9(5):e97613. doi: 10.1371/journal.pone.0097613 (PMC4029741; doi:10.1371/journal.pone.0097613)
Supplement: Table S1 — General characteristics of patients diagnosed with acute/chronic pancreatitis or pancreatic cysts (data presented as means ± SD or median [interquartile range]). (PDF) [file pone.0097613.s002.pdf]

Table S1. General characteristics of patients diagnosed with acute/chronic pancreatitis or pancreatic cysts (data presented as means ± SD or median [interquartile range]).

Parameters	"other pancreatic diseases" group
Age (years)	47 ± 10*#
Gender (M-men/W-women)	31-M/14-F
BMI (kg/m^2)	25.91 ± 4.12
RBC (x10^{12} cells/L)	4.27 ± 0.83
Hb (g/dL)	12.73 ± 1.80*
Platelets count (x10^9 cells/L)	313 ± 141
WBC count (x10^9 cells/L)	8.49 ± 3.62
CRP (mg/L)	23.12 [4.28; 97.90]*#
CA19.9 (U/mL)	19.81 ± 16.92#

BMI – body mass index RBC – red blood cells Hb - hemoglobin

WBC – white blood cells CRP – C-reactive protein

*P<0.01 (vs control group)

#P<0.01 (vs cancer group)
