# Supplementary material for: Selected Cytokines in Patients with Pancreatic Cancer: A Preliminary Report
Source: PLoS One. 2014 May 21;9(5):e97613. doi: 10.1371/journal.pone.0097613 (PMC4029741; doi:10.1371/journal.pone.0097613)
Supplement: Table S2 — Cytokine and C-reactive protein levels in pancreatic cancer patients, subdivided into groups according to the Tumor-Node-Metastasis (TNM) staging of malignancy (presented as means ± SD or medians [interquartile range]). (PDF) [file pone.0097613.s003.pdf]

Table S2. Cytokine and C-reactive protein levels in pancreatic cancer patients, subdivided into groups according to the Tumor-Node-Metastasis (TNM) staging of malignancy (presented as means ± SD or medians [interquartile range]).

**Subgroup/ Parameter**	**Early/Resectable* (n = 6)**	**Locally advanced* (n = 10)**	**Metastatic* (n = 27)**
IL-6 (pg/mL)	8.75 [5.75; 11.55]	10.11 [3.35; 16.84]	9.88 ± 5.32
IL-8 (pg/mL)	47.85 [31.03; 64.21]	65.60 [52.13; 78.56]#	80.45 ± 28.55#
IL-10 (pg/mL)	12.17 [6.62; 18.73]	12.46 [4.67; 20.88]	14.01 ± 9.15
IL-23 (pg/mL)	19.88 [7.14; 32.01]	22.31 [16.24; 30.38]	22.19 ± 7.90
TNFα (pg/mL)	11.35 [9.98; 12.72]	10.09 [5.86; 14.34]	14.55 ± 10.68
CRP (mg/L)	11.7 [4.8; 15.9]	17.6 [5.1; 29.3]	19.0 [5.4; 64.9]

* “Early/Resectable” patients were classified as TNM Stage 1 or 2; “Locally advanced” represented TNM Stage 3 and “Metastatic” TNM Stage 4

CRP – C-reactive protein

IL – interleukin

TNFα – tumor necrosis factor alpha

# $p < 0.05$ (vs “Early/Resectable” group)
