# Supplementary material for: Selected Cytokines in Patients with Pancreatic Cancer: A Preliminary Report
Source: PLoS One. 2014 May 21;9(5):e97613. doi: 10.1371/journal.pone.0097613 (PMC4029741; doi:10.1371/journal.pone.0097613)
Supplement: Table S3 — Levels of examined cytokines in patients with other malignancies (NETs and SPTs) together with their statistical comparison (medians [interquartile range]). (PDF) [file pone.0097613.s004.pdf]

Table S3. Levels of examined cytokines in patients with other malignancies (NETs and SPTs) together with their statistical comparison (medians [interquartile range]).

**Cytokine** [pg/ml]	**other malignancies group**	**p vs control**	**p vs cancer**
IL-1	3.08 [2.52 ; 3.44]	0.64	0.48
IL-6	7.70 [3.66 ; 9.95]	0.08	0.82
IL-8	56.32 [13.7 ; 89.18]	0.48	**0.05**
IL-10	6.50 [5.10 ; 8.38]	0.30	0.60
IL-12	3.50 [3.00 ; 4.55]	0.20	0.35
IL-17	115.7 [105.5 ; 130.0]	0.65	0.97
IL-23	72.74 [57.88 ; 83.22]	**0.007**	**0.002**
TNFα	6.30 [5.36 ; 8.34]	0.63	**0.03**
G-CSF	36.40 [29.85 ; 38.65]	0.81	0.32

IL – interleukin

p – level of significance

TNFα – tumor necrosis factor alpha

G-CSF – granulocyte-colony stimulating factor
