# Supplementary material for: Selected Cytokines in Patients with Pancreatic Cancer: A Preliminary Report
Source: PLoS One. 2014 May 21;9(5):e97613. doi: 10.1371/journal.pone.0097613 (PMC4029741; doi:10.1371/journal.pone.0097613)
Supplement: Table S4 — Coefficients of correlations between absolute numbers of circulating bone marrow-derived stem cells' populations and systemic levels of examined cytokines in patients with pancreatic adenocarcinoma (n = 8). (PDF) [file pone.0097613.s005.pdf]

Table S4. Coefficients of correlations between absolute numbers of circulating bone marrow-derived stem cells` populations and systemic levels of examined cytokines in patients with pancreatic *adenocarcinoma* (n = 8).

**stem cells population/parameter**	**IL-6**	**IL-8**	**IL-10**	**IL-23**
**Pancreatic cancer patients**				
**VSEL**	NS	NS	NS	**-0.65#**
**MSC**	**0.96***	**0.98***	**0.71#**	**-0.62#**
**HSC**	NS	NS	NS	NS
**EPC**	NS	NS	NS	NS

#P<0.05 *P<0.005 P – level of significance NS – not significant

VSEL – very small embryonic-like stem cells MSC – mesenchymal stem cells

HSC – hematopoietic stem cells EPC – endothelial progenitor cells
